# The cultural and structural influences that ‘hide’ information from women diagnosed with breast cancer in Ghana: an ethnography

**DOI:** 10.1186/s12905-021-01502-2

**Published:** 2021-10-15

**Authors:** Linda Serwaa Agyemang, Claire Foster, Chris McLean, Deborah Fenlon, Richard Wagland

**Affiliations:** 1 Health Sciences, Faculty of Environmental and Life Sciences, University of Southampton, University Road, Southampton, SO17 1BJ UK; 2 Department of Nursing, College of Human and Health Science, University of Swansea, Swansea, UK

**Keywords:** Patient-provider relationships, Socio-cultural factors, Language barriers, Participant observation, Qualitative interviews, Ethnography, Decision-making

## Abstract

**Background:**

Socio-cultural factors may influence the uptake of breast cancer treatments. This study aimed to explore these socio-cultural influences on treatment decision-making for women in Ghana.

**Method:**

An ethnographic approach was adopted. Observation was conducted of women newly diagnosed with breast cancer, nominated relatives, nurses and doctors at a breast clinic in Ghana. Semi-structured interviews followed participant observation. Thematic analysis was employed.

**Findings:**

Over 16 weeks (July 2017–November 2017), 31 participants were observed and 29 took part in semi-structured interviews. Three overarching themes were identified: (1) unequal power relationships; (2) Language barriers and (3) structural constraints. Following a breast cancer diagnosis, essential information necessary for treatment decision making is ‘hidden’ from women due to an unequal patient-provider relationship. Patients acknowledged cultural behaviours of deference to experts. Doctors deliberately misrepresented treatment information to women to encourage them to undergo surgical treatment. Structural issues such as the lack of privacy during consultations hindered quality patient engagement with decision-making. High treatment costs and the lack of resources to assist women with fertility after treatment impeded open discussions around these issues. Language barriers included a lack of terms in the local Twi language to explain cancer and its treatment. There was also an absence of appropriate information materials.

**Conclusion:**

Findings highlight the need for health professionals to be aware of the socio-cultural factors that limit access to quality information which is needed for informed treatment decision making. Policies that aim to provide adequate logistics; increase staffing levels; improve staff cultural awareness training and remove financial barriers are recommended.

## Background

Breast cancer is the most frequently diagnosed cancer among women worldwide. In 2018, the global estimates of breast cancer were 2.1 million new cases [1], with approximately 50% in developing countries [2]. In Ghana, and comparable low- and middle-income countries in sub-Saharan Africa, over 60% of women have advanced (stage 3 and 4) breast cancer at diagnosis [3, 4] compared to approximately 33% in higher income countries like the USA [5].

Estimates for the five-year survival rate for breast cancer in Ghana is 39% [6] compared to 91% in the USA [7]. However, the absence of a Ghanaian national cancer registry means rates may be under-reported.

The influence of culture on health behaviours has long been documented in the literature [8]. Previous studies have identified prevalence of traditional healing modalities as contributors to delayed diagnosis and treatment among Ghanaian women [9, 10]. Socio-cultural factors that may influence women’s decision making include cultural beliefs that breast cancer has supernatural causes, that mastectomy inevitably leads to death; that a woman cannot be ‘complete’ following mastectomy; and the centrality of God to healing [10–14]. Stigma attached to breast cancer diagnosis and mastectomy as well as financial constraints have been shown to delay treatment [13, 15].

Studies conducted in Africa highlight how socio-cultural factors inhibit the timely uptake of breast cancer treatment [9–15]. However, little is known about how these factors shape the involvement of women in the decision-making processes. The aim of this study is to explore and understand how the cultural context influences the decision-making process for women attending a breast clinic in southern Ghana.

## Methodology

An ethnographic approach was adopted. Data collection involved observation of the interactions between clinicians and women and their relatives as they navigated the breast clinic and received their diagnosis and treatment recommendations. Semi-structured interviews then explored what had been observed with participants in greater depth.

### Setting

The study took place in a public teaching hospital in Southern Ghana that provided comprehensive breast cancer management. The hospital is the regional centre for cancer treatment and breast cancer is the most common cancer presented there [16]. As a referral point for the Northern, Middle, Western and Central parts of the country, the clinic is accessed by a heterogeneous group of people.

### Sample

Purposive maximal variation sampling was used to sample women whose treatment had curative intent (up to stage 3 breast cancer); women-nominated family members; nurses; and doctors at a breast clinic in southern Ghana. The study focused on two time points, firstly at formal diagnosis (where core biopsy reports were available to patients), and secondly at a tumour board meeting, where ‘women’s treatment options were discussed’.

### Data collection

Participant observation was conducted by LA (July 2017 to November 2017). Observation concentrated on the information women received and the dynamics between healthcare professionals (HCPs), women with breast cancer and their family members (i.e., seating arrangements, who led the discussions, verbal and non-verbal communication).

Focused observations mostly lasted between one and four hours on a clinic day and continued until data saturation when no new insights emerged. Field notes were kept during participant observation and women and their nominated relatives were interviewed following their tumour board meeting, when treatment options were discussed. Some HCPs were interviewed half-way through participant observation (to allow for concurrent preliminary data analysis) and others towards the end. All participant interviews followed an interview guide (Appendix 2–4).

Interviews were conducted mostly in Twi, a local Ghanaian Language, unless participants chose to speak in English. For the patients and their family members, interviews were conducted either at the clinic, their homes or workplaces, and clinicians’ interviews were conducted in the clinic setting [17, 18]. Interviews were audio recorded and field notes were recorded describing conversations, descriptions of the context, and personal reflections. Interview audio recordings were first transcribed verbatim in Twi and later translated into English by LA, with the accuracy of translations validated by a local expert. Each participant was assigned an exclusive pseudonym [19].

### Data analysis

Thematic analysis was conducted on data from both the observations (field notes) and interviews using NVivo 12 (computer assisted data analysis software) [19, 20].

### Reflexivity

LA is a registered nurse and a native Twi speaker, but she was not known to the study participants before undertaking the study.

### Ethics

Ethical approval was obtained from Kwame Nkrumah University of Science and Technology (Reference: CHRPE/AP/393/17) and University of Southampton (Ethics ID: 26,346). Participants were provided with information sheets outlining the study aim and research process after which written consent was obtained. Participants were informed of their right to refuse participation [21].

### Findings

A total of 89 h of participant observation was conducted at the breast clinic over a 40-day period. 31 participants were observed: 16 women with breast cancer stage 1–3; five self-nominated family members; ten HCPs, including five nurses; and five doctors. Of the 31 participants (Table 1), 29 were interviewed and two HCPs were not interviewed following observation due to time constraints and work demands.

**Table 1:** Characteristics of participants

Participant	Characteristics	Category of characteristics	Number of participants
Patients (n = 16)	Age (years)	20–30	1
		31–40	3
		41–50	4
		51–60	3
		61–70	2
		71–80	2
		81–90	1
	Number of children	None	2
		1–3	9
		4 or more	5
	Ethnicity	Asante	6
		Sefwi	1
		Akyem	1
		Bono	2
		Wassa	1
		Kwahu	1
		Akuapim	1
		Fanti	1
		Dagarte	1
		Frafra	1
	Education	No formal education	3
		Primary or elementary	9
		Secondary	1
		Tertiary	3
	Monthly income* of patient participants (US Dollars)	None	2
		Irregular	9
		85–170	4
		171–499	0
		500–850	1
	Breast cancer stage	Stage 2	7
		Stage 3	9
	Recommended treatment	Breast conservation, adjuvant	1
		Mastectomy	1
		Mastectomy, adjuvant	3
		Neoadjuvant, mastectomy, adjuvant	11
Relatives/informal carers (n = 5)	Age	Median 32 years; Range 23 years	5
	Ethnicity	Asante	2
		Akyem	2
		Sefwi	1
	Education of relative participant	Primary or elementary	3
		Secondary	1
		Tertiary	1
	Monthly income of relative participants (US Dollars)	Irregular	3
		50–99	1
		100–199	0
		200–250	1
Healthcare professionals (n = 10)	Professional/clinical role	Consultant general surgeon	2
		Resident	3
		Adult general nurse	4
		Nurse/Midwife	1
	Years in service	10 or more	3
		> 5 < 10	3
		Up to 5	4

*Daily minimum wage in Ghana is approximately Two US Dollars (Africa Labour Research and Education Institute, 2021)

### ‘Hidden information’

During patient-clinician interactions, important information about the disease stages and treatment, (expected outcomes, risks, and benefits) were rarely provided. For instance, when patients and relatives described their views on diagnosis and treatment, most reported they received little information about the disease and treatment. A few commented:

“when I came, I have not been told much, so cancer I don’t know anything” (Foriwaa, 48 years)

“Umm they did not say much, they just said they will give drugs and then they will do operation for her” (Maame Saa’s son, 29 years).

This perspective was corroborated by observations of the organised tumour board meetings where patients and relatives were invited, but are then asked to wait outside, whilst HCPs discuss their treatments in their absence. As patients are rarely involved in treatment discussions, they often report feeling disempowered and have little knowledge about their disease or the treatment options available to them.

“I was expecting the doctor to talk to me when I do the test, but he did not say anything…mmm, no one really tells me anything, even the day I went to the [Tumour Board] meeting, we sat waiting for a long time, it was later the nurse came to tell me I will be coming to oncology for some injection, so I have not been able to talk to anyone, I don’t really know what the treatment will involve” (Maame Tawia, 52 years).

The ‘hidden’ nature of information about breast cancer and its treatment was influenced by three main themes: unequal power relationships, language barriers and structural constraints (see Table 2).

**Table 2:** Themes and sub-themes relating to ‘hidden’ information

Theme	Sub-theme
Unequal power relationships	(i) Passivity and deference to HCPs (ii) Deliberate miscommunication of treatment intent
Language barriers	
Structural constraints	(i) Under resource of the breast clinic (ii) Lack of discussion about costs (iii) Limited discussions about fertility

### Unequal power relationships

#### Passivity and deference to HCPs

Patients often reported they would rather defer to the greater expert knowledge of HCPs and were unlikely to ask questions even if they did not understand something.

“Umm madam, I know if anything at all it is doctors who are going to do everything, so I did not ask question” (Lydia, 45 years).

Their perceived need to acquiesce to authority therefore reinforced their silence. Patients feared that asking HCPs questions or raising concerns may be interpreted as challenging the authority, competence and professionalism of staff and cause them to receive poorer care.

“so, I don’t do anything that will sound that I am challenging them and be asking too much questions, so I need to come down so they also take care of me” (Maame Abigail, 68 years)

#### Deliberate miscommunication of treatment intent

HCPs were aware of the misconceptions and the cultural beliefs regarding the spiritual origins of cancer which prevented women from proceeding with treatment. They sometimes used ambiguous rather than specific terms, such as, ‘operation’ rather than ‘mastectomy’, to hide the actual treatment intent and to influence the behaviour of women and prevent them from refusing treatment.

“using ‘operation’ is intentional, we don’t want them to run away” (comment from a doctor as recorded in the Field note).

“he just called one after the other and said something briefly that I will have drugs after they will do operation and remove it for me, … he did not say he will cut the breast, he said he will remove the lump” (Foriwaa, 48 years).

The deliberate miscommunication of the proposed treatment effectively ‘hides’ such information from patients. Other HCPs, however, did not support this approach and encouraged their colleagues to be open about their intended treatments.

#### Language barriers and limitations in the local language to describe cancer

The diagnosis provided to women was defined as ‘cancer’ or ‘kokoram’. The word ‘cancer’ is an English word and although English is spoken in Ghana as a second language, it is learned during formal education. Hence, people without formal education may have difficulty understanding what ‘cancer’ means. ‘Kokoram’ is the local Twi word for cancer, but there are over 40 languages spoken in Ghana which all are limited in the words they have to describe cancer. As women who present to the clinic are ethnically diverse, this lack of appropriate language terms constitutes a barrier to their understanding.

Secondly, there are also no words to explain certain concepts (types of breast cancer; cancer staging; chemotherapy; radiotherapy; hormonal; and biologic therapies). The lack of definitive words was evident during interactions between patients and HCPs. For example, it was observed that chemotherapy was referred to as ‘cancer injections’ and radiotherapy as ‘heat’ by staff. The limited concepts to explain cancer and treatment limits the understanding amongst patients and constrains decision making. A third issue relating to language is the content of posters at the clinic which are largely in English and inaccessible to patients and relatives with limited formal education. Furthermore, investigation results are coded in medical terms and so people with little or no health literacy background may find them challenging to understand. One patient relative commented:

“Umm they have not said anything whether it is this or that. [The doctor] said they will give drugs and then they will do operation for her, but the tests we went to do, I read the report and it says there is nothing but the histology report too when I read, I could not really make anything out” (Maame Saa’s son, 29 years).

Overall, the effect of the limitations of language and terminology in this context limits access or ‘hides’ quality information about breast cancer and treatment from patients and relatives.

### Structural barriers that reinforce gaps in knowledge about the disease and treatment

#### Under resourced clinic

Limited expertise in breast cancer amongst HCPs, as well as the inadequate number of staff and consulting rooms, meant the capacity for effective patient-clinician interactions was also limited. Two nurses mentioned that they were unable to engage patients and provide them with the necessary information due to time constraints and workload.

“we should talk to them and give information for them to understand, but we don’t normally or regularly do it because of time or workload” (Nurse C)

The doctors also mentioned the problem that ‘the job is plenty’ [ie demanding] for them because they are not breast specialists but general surgeons, which meant they had other commitments from other surgery clinics.

“here we are not breast specialists, if we do breast today, tomorrow we are doing hernia, the next day goitre, the next day stomach cancer so it does not make the focus to be solely on the breast…” (Dr C)

Many of the patients mentioned that following a life-threatening diagnosis, they reacted with sadness, confusion and fear, which meant they were unreceptive to information or unable to ask for clarification at the time of diagnosis. Hence, two patients reported they would have liked to have a follow-up contact with staff, which was absent.

“on that day, when the doctor told me about the results that it is cancer, hmm, all that I was thinking about was I am coming to die, therefore, all the things he said afterwards I did not hear anything at all. I heard him talking, but none made any meaning to me because all that kept coming back to my mind was the word cancer…so when I went home, I felt there is no telephone number to call but I wanted more information later on…” (Adwoa, 32 years).

Both patients and doctors were not able to discuss sensitive information due to frequent interruptions and often the presence of other patients in the same consulting room. This issue was reported in some of the interviews:

“Mmm, the people in the consulting room were many, they keep coming to interrupt and ask him something and I was thinking me too I want to ask this question, but these people keep coming to the doctor to show him things, so the doctor did not get time, he could not get any time” (Maame Tawia, 52 years)

The lack of quality engagement with clinicians left patients feeling disempowered and lacking understanding and information of their condition and treatment options.

#### Lack of discussion about costs

Both HCPs and patients acknowledged that investigation costs are one of the biggest challenges to patients adhering to treatment. HCPs therefore found discussions with women about treatment costs uncomfortable because they knew they were often unaffordable. The doctors often avoided such discussion if possible and delegated this task to the nursing staff.

“…Now what I am going to do is to write some investigation request for you to do. You will need to have chest CT, abdominal ultrasound, …. Do you have any questions?” Sara looks at doctor D as he talks and then replies, “how much do the things you are asking me to do cost?” Dr D (looks at Sara), “I do not know but the nurses may be able to assist you”. Field note

Similarly, the nurses do not divulge this information either, but rather ask patients to check at the various departments where the investigations are performed. Women therefore navigate the clinic with limited information on the cost of requested investigation or treatments.

#### Limited discussion about fertility issues

In Ghana, breast cancer is usually diagnosed among women who are young, some of whom are yet to get married or have children. HCPs acknowledged that childbearing is highly valued in Ghanaian society and reported that women often decided against undergoing treatment due to their concerns about its impact on fertility.

“childbearing is important in this setting, so it is also a problem we face…some don’t come back when we tell them treatment can delay or prevent childbearing and we don’t have resources to help women to conceive after treatment in this hospital” (Doctor E)

A few of the women raised concerns about not knowing how their fertility will be impacted by treatment.

“I was thinking about childbearing, that what if, so I am thinking will the chemo affect my ability to have other children. … but it seems the way the doctors were asking me, do I have only one child, so it seems there is a question mark somewhere that they don’t want to bring it out” (Natasha, 28 years).

The lack of honest discussion meant patients’ concerns around fertility were not adequately addressed. This left gaps in their knowledge which could create avenues for misinformation and a consequent loss of women to follow-up.

“So, if women are not able to ask questions and we also don’t give much information, it contributes to us losing them because there is a lot of misinformation. What I will say lot of gaps, so they begin to fill in the gaps themselves with wrong information, … then if that information happens to be wrong then that is it, we lose them” (Dr D).

## Discussion

These findings demonstrate that following a diagnosis of breast cancer, cultural and structural factors ‘hide information’ regarding treatment options for Ghanaian women which is detrimental to their decision making. A culture of hierarchy and deference ensured women were passive and deferred to HCPs as the ‘experts’ regarding their treatment decisions, a finding in previous research [22]. The limited discussions between patients and HCPs left patients susceptible to misleading information from other sources. Anarado et al. [23] had a similar finding, where lack of information and support from nurses created gaps that threatened adherence to prescribed chemotherapy courses among Nigerian women with breast cancer.

High levels of deference to authority figures reflects the hierarchical nature of Ghanaian society [24]. Within social settings, seniors are viewed as wise and should be consulted by others when making decisions [24, 25]. It is believed that as doctors have acquired wisdom and expert knowledge from education [24], patients should defer to their judgement and allow doctors to make decisions that affect patients’ interests [24].

Unlike the shared decision-making model practised in many western countries [26], the socially hierarchical practices in Ghanaian society limits patients’ active involvement and participation in discussions about their treatment options. A finding unique to this study was the practice amongst HCPs of consciously misrepresenting the treatment intent to women with the aim of influencing their decisions. It was evident in the study that the hiding of treatment intent (especially mastectomy), often until the last moment before the patient was due to have surgery, could be shocking to the woman. It could also mean the woman may not have had the chance to grieve [27] the potential loss of the breast, which might deter her from persevering with the surgery. HCPs should be aware of the constraining effects of hierarchical practices and encourage patients to express their opinions and preferences, and should also minimise interruptions at the clinic.

In addition to cultural factors, resource constraints also limited the ability of women to participate in the treatment decision-making process. Previous studies in Ghana [15] and Nigeria [11] have also shown limited resources influenced breast cancer treatment uptake. In the present study, the teaching hospital where the breast clinic was located was a referral hospital for malignant cases for over 10 million people living in the northern, middle and some southern parts of the country [28]. The clinic can hence be overwhelmingly busy for the limited number of staff to effectively engage with patients.

The World Health Organization has emphasised the suffering of patients with cancer in developing countries due to the limited resources [29]. For example, patients in this study were not given any specialist attention, particularly with regards to their psychological concerns and emotional needs [12]. Language also represented a barrier to understanding [30]. Although both HCPs and patients communicated in the same language (despite some differences in ethnic background), there were nevertheless limited concepts in the local language to explain breast cancer and the various treatment approaches. In the present study, three-quarters of the 16 women had either basic or no formal education at all: a proportion similar to that reported in the census report from the Ghana Statistical Survey in 2010 [28]. In the census report, the literacy rate amongst women 15 years and older was 49.8% [28]. Limited formal education may impact on effective patient-clinician interaction and can limit patients’ understanding of medical matters, a finding in previous studies in Nigeria [31] and Ghana [32]. These linguistic factors construct a further barrier which ‘hides’ information from women that could otherwise empower them. It is likely that women will seek to fill the consequent gaps in their knowledge with more easily accessible misinformation and misconceptions, such as ‘mastectomy equals death’, that could be harmful for their decision making.

This issue has implications for policymakers and clinicians. At the level of the clinic, HCPs would require more training to increase their awareness about the issues impacting breast cancer treatment decision making. Although some of the issues (financial barriers, formal education; accessibility to health information and language) are outside the control of HCPs, they nevertheless need to be aware of the vulnerabilities of patients who visit the clinic and create an enabling atmosphere to engage patients and explore their concerns. HCPs could further collaborate with patients and survivors of breast cancer to provide enhanced supplementary information in appropriate formats (e.g., videos and pictures). Policy makers should allocate more funding towards cancer care and research as advocated by the WHO [33].

The study’s strength is derived from a prolonged engagement in the field. In addition, the study involved more than one group of participants (i.e., patients, families, HCPs), which facilitated triangulation and a broader understanding of the different contextual aspects impacting breast cancer treatment uptake. The status of the researcher in the field as both an insider and outsider (a Ghanaian nurse but not a member of the clinic team) were a strength for this study, which involved an understanding of the common local language and ensured relationships of trust could develop with study participants.

Despite these strengths, recruitment from a single hospital was a limitation for this study as patients who were not able to attend this hospital could not be included.

## Conclusion and future research

Sub-optimal treatment is a challenge to breast cancer control in Ghana. Cultural and structural factors ‘hide’ essential information necessary for effective decision making, which may reduce the uptake of treatment. The findings of this study could therefore serve as a needs’ analysis for a decision support interventional study in the near future.

## Data Availability

Due to confidentiality issues, dataset analysed for this study can be made available (from corresponding author) upon request.

## Appendix 1

### Observation guide

**Table:**

Participants involved	Physical space:
Date	Time
Activity	Goal
Objects	Non-verbal behaviours

## Appendix 2

### Interview guide for patient participant

#### Background information

**Table:**

Age	Marital status	Menstruation status
Parity	Ethnicity	Place of residence
Occupation	Income	Religion
Educational level		

1. Can you describe your thoughts about what the doctor and the nurse talked to you concerning breast cancer and treatment? Prompts: How clear are you about the pros and cons that are important to you concerning the treatment? What information did you want? Did you ask questions or clarification? Tell me more?
2. What concerns do you have about the disease and treatment? Prompts: Who did you share these concerns with? Tell me more?
3. Ask about behaviour the researcher observed; ask about meaning of behaviour observed; how does this experience influence your treatment decision?

## Appendix 3

### Interview guide for relative participant

**Table:**

Age	Ethnicity
Relationship to patient	Educational level
Income	

1. Can you share with me your views about breast cancer and treatment?
2. Can you tell me your concerns about breast cancer treatment (prompt: How do your views and concerns about breast cancer and treatment influence your support to the woman i.e. informational support, instrumental support, emotional support?)
3. How do you describe your role in the woman’s treatment decision making?

## Appendix 4

### Interview guide for HCP participant

#### Background information

**Table:**

i. Professional background	ii. Years of practice at the breast clinic

1. Can you describe your role during breast cancer diagnosis to women attending this clinic? (prompt: how do you provide diagnosis)
2. Can you describe your role during treatment recommendation to women with breast cancer? (prompt: how do you offer treatment recommendation)
3. Can you describe what patients’ views are regarding breast cancer and treatment? (prompt: in what ways does this influence patient decisional support)
